# Transferring Learned Behaviors between Similar and Different Radios

**DOI:** 10.3390/s24113574

**Published:** 2024-06-01

**Authors:** Braeden P. Muller, Brennan E. Olds, Lauren J. Wong, Alan J. Michaels

**Affiliations:** 1 Virginia Tech National Security Institute, Blacksburg, VA 24060, USA; braedenm@vt.edu (B.P.M.); beolds@vt.edu (B.E.O.); 2 AI Lab, Intel Corporation, Santa Clara, CA 95054, USA

**Keywords:** transfer learning, radio frequency machine learning (RFML), automatic modulation classification (AMC), specific emitter identification (SEI), captured data

## Abstract

Transfer learning (TL) techniques have proven useful in a wide variety of applications traditionally dominated by machine learning (ML), such as natural language processing, computer vision, and computer-aided design. Recent extrapolations of TL to the radio frequency (RF) domain are being used to increase the potential applicability of RFML algorithms, seeking to improve the portability of models for spectrum situational awareness and transmission source identification. Unlike most of the computer vision and natural language processing applications of TL, applications within the RF modality must contend with inherent hardware distortions and channel condition variations. This paper seeks to evaluate the feasibility and performance trade-offs when transferring learned behaviors from functional RFML classification algorithms, specifically those designed for automatic modulation classification (AMC) and specific emitter identification (SEI), between homogeneous radios of similar construction and quality and heterogeneous radios of different construction and quality. Results derived from both synthetic data and over-the-air experimental collection show promising performance benefits from the application of TL to the RFML algorithms of SEI and AMC.

## 1. Introduction

Signal identification (SI) is the task of determining critical descriptors of a transmission, such as technology, modulation type, interference source or interference-free channels, or even emitter identity from only the characteristics of received signals [1,2]. This task originally found use for military and regulatory applications, such as electronic warfare, spectrum awareness, and spectrum monitoring but has recently garnered interest in the commercial space for intelligent radios [3]. The widespread capability of RF measurement but lack of reliable expert-defined algorithms for this task makes signal identification a logical target for radio frequency machine learning (RFML)-based classification algorithms [1].

Automatic modulation classification (AMC) and specific emitter identification (SEI) are two of these SI tasks, which involve determining the modulation scheme and the identity of a received signal, respectively [4,5]. RFML techniques for these tasks have shown great success in classifying signals based on unique features that are consistently present in signals of particular classes [6,7,8,9]. However, these models are usually created based on data generated from only one type of receiver and, therefore, struggle to generalize when applied to data generated with different hardware under different conditions [10,11]. In order to apply the existing classification algorithms to new platforms, the models must be retrained on data specifically created for the new platform [12].

The process of collecting new data for a desired platform and retraining a model can be time-consuming and expensive. Many examples of algorithms across a variety of domains suffer from these limitations, which stem from a strong reliance on training data. Examples include how discriminative learning methods for natural language processing, when trained on data from only one domain, fail to generalize across different domains [13], and training machine learning models for real-time strategy games using data from individual game scenarios can result in a variety of shortcomings [14]. The weaknesses presented in these works are analyzed, then overcome, through a concept known as transfer learning [15].

Transfer learning (TL) allows for learned information from a source domain and learning task to be applied to a different target domain or learning task [16,17]. This lets the first model’s learned information be re-used, tuned, and added to the training set of the second model. The benefits of transfer learning can be seen in relation to the above examples, where the work reported in [13] correlates features from different domains to increase model accuracy and the method described in [14] applies knowledge learned from a source game scenario to a target scenario to improve model performance. In a similar manner, TL can be applied to RFML classification algorithms in an effort to reduce the amount of additional hardware and platform-specific retraining that must be performed or simply to boost performance.

Numerous attempts to apply similar improvements to the RFML techniques of SEI and AMC using TL have been studied in the literature. Existing SEI works include a large variety of applications of TL to improve results. One such application is in the use of TL to perform feature reconstruction for SEI in an effort to improve robustness to signal-to-noise ratio (SNR) variations [18]. Another approach can be found in the utilization of TL to improve data clustering and resampling to create datasets that are less impacted by source-domain biases [19]. Additionally, TrAdaBoost, an inductive TL method, has been applied to Support Vector Machines (SVMs) used for SEI to filter out data from the source domain that are not applicable to the target domain through weight adjustments, allowing for the newly trained model in the target domain to utilize only relevant data from the source domain [20]. TL has also been applied to the use of Long Short-Term Memory (LSTM) networks for SEI in order to improve model learning efficiency by helping the model to learn the prediction function in the target domain [17]. CNNs created for SEI have also benefited from the application of TL through the use of Knowledge Distribution (KD), which is an algorithm for transferring learned knowledge from a large model to a small model by designing and minimizing the loss function gathered from the labels of the teacher model and training dataset [21]. A comparison of some previous works utilizing TL for RFML classification tasks is presented in Table 1.

In regards to AMC, transfer learning has been used in a variety of efforts to improve algorithms of various structures, such as in seeking to reduce domain shift in adversarial architectures [28] and improving CNN-based AMC models’ ability to classify low-quality datasets through the transfer of learned features from datasets with varying SNR values [29]. TL has also been applied to semi-supervised AMC models to reconstruct unlabeled modulation signals and apply that knowledge to a CNN for classification [30]. Additionally, TL has been applied to improve model retraining efficiency of CNNs by removing the need to consider repeated modulation schemes that were already considered in the previously trained model and including carrier-phase compensation data from a previously trained model to allow for models to be trained on datasets lacking carrier-phase compensation data [31]. TL can also be seen being used in Deep Learning (DL) methods to perform AMC by starting with pre-trained ResNet-50 and Inception ResNet V2 models and training them with input images representing 1000 classes to create constellation density matrices the performance of AMC [32,33]. Each of these applications of TL to the RFML domain has shown success in increasing model performance or decreasing the burden of model retraining and has, thus, provided a strong argument for further implementing TL in other RFML applications [34].

This work intends to assess the viability of transfer learning in an RFML context between similar radios and those of comparably lower performance. This is done by evaluating the performance of AMC and SEI models when trained and tested on an RF dataset captured from one type of radio, then comparing the results to those gathered from a TL model that augments the prior model with data from another type of radio of relatively lower quality. The reverse of this is also evaluated, namely transferring from a relatively lower-quality radio to a relatively higher-quality radio. By comparing the accuracy of the baseline model with no transfer learning to data obtained by transferring from high-to-low and low-to-high quality radios, a comprehensive analysis of the studied models’ abilities to generalize across different types of radios can be determined. This analysis allows for conclusions to be drawn relating to the effectiveness of models both with and without the TL approach by showing how effectively they can perform in the presence of varied emitters, thus increasing security and spectrum situational awareness in the RFML domain.

This paper begins with the evaluation framework and methodology in Section 2, emphasizing models and CNN training processes that apply to both synthetic and real-world collected data. The hardware-based data collection setup and a description of the ML model trained for both AMC and SEI are presented in Section 3. Section 4 then describes the results and takeaways, comparing the performance of synthetic (simulation-based) and real-world data sources, as well as noting disparities in how radios of differing quality perform with RFML algorithms. Lastly, conclusions and recommendations for future work are discussed in Section 5.

**Figure 1:**
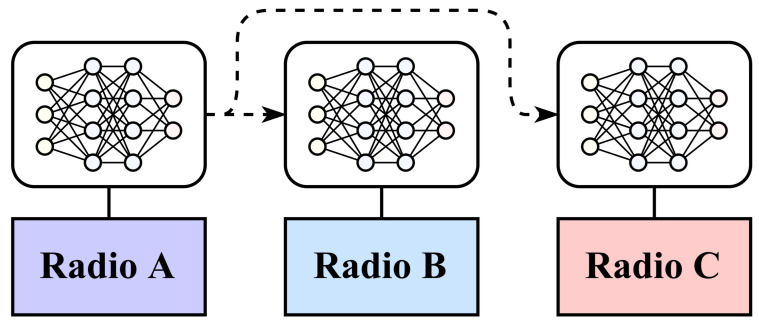
Model weights, biases, and states for a model trained on one radio are re-used for classification on the other radios.

Transfer learning performance is evaluated based on real-world captured datasets from three radios labeled A, B, and C. Radios A and B are the same model of radio and are identical in specifications, and radio C is of comparatively lower quality. The same ML architecture, as presented in Section 2.2, is used for every trial for both AMC and SEI classification. In this circumstance, transferring a radio from one model to another consists of re-using the weights, biases, and long short-term memory (LSTM) cell states of a model trained on one dataset as a starting point for the training of a model for a new radio, as shown in Figure 1.

## 2. Methodology

### 2.1. Dataset Segmentation

**Figure 2:**
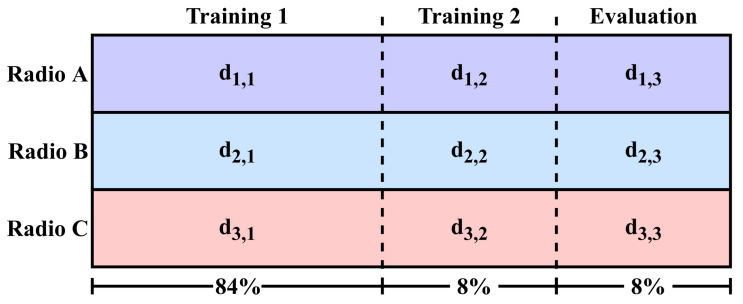
Division of datasets for training, transfer, and evaluation of RFML models. Each segment is labeled $d_{x,y}$, where *x* labels the source radio (A = 1, B = 2, and C = 3) and *y* labels the division (phase 1 training = 1, phase 2 training = 2, and evaluation = 3).

Datasets are divided into the following three primary segments: 84% training phase 1, 8% training phase 2, and 8% evaluation, as shown in Figure 2. Each training phase is further sub-divided into 91% training and 9% validation sets. For both AMC and SEI, the training phase consisted of 12,000 training examples per class (60,000 total for AMC and 720,000 total for SEI) and 1200 validation examples per class (6000 total for AMC and 72,000 total for SEI). This was based on previous work that used this model architecture that had observed that gains in AMC classification accuracy plateaued past 10,000 examples per class [35].

As a baseline for analysis, performance is measured when trained on training phases 1 and 2 of a dataset (as shown in Figure 2), then evaluated on the evaluation portion of the same dataset, denoted as $\left(d_{x,1},d_{x,2}\right)\to d_{x,3}$. To assess transfer learning feasibility for a particular scenario, performance is measured when trained on training phase 1 of the source radio, then further trained on training phase 2 of the destination radio and evaluated on the evaluation set of the destination radio, namely $\left(d_{x,1},d_{y,2}\right)\to d_{y,3}$. The generality of this transferred model is tested when performance is also evaluated on the source evaluation set, namely $\left(d_{x,1},d_{y,2}\right)\to d_{x,3}$. Performance in each trial is presented in a *confusion matrix*, which shows how frequently an example of a particular radio or modulation scheme is classified as each label.

### 2.2. Classification Architecture

**Figure 3:**
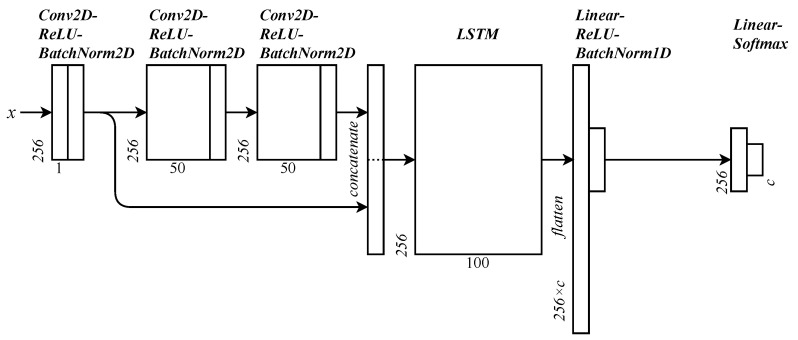
The structure of the CLDNN used for both AMC and SEI classification. The number of possible classes is represented by the variable *c*.

The chosen architecture trained for each RFML model is a convolutional, long short-term memory (LSTM) deep neural network (CLDNN) [36]. This architecture is similar to a (dense) deep neural network (DNN) with added convolutional layers to assist in identifying time-varying patterns and LSTM layers to help mitigate the negative effects of vanishing gradients. The network has 1 convolutional layer whose output is both fed into the next set of 2 convolutional layers and concatenated to the output of these layers. This combined string is fed into an LSTM network, whose output is flattened and fed into a linear layer. All layers up until this point are followed by ReLU activation functions and batch normalization layers. The final layers are a linear layer and a Softmax output for each class. The structure of the network is illustrated in Figure 3. This architecture was chosen because it has historically shown high performance for AMC and SEI tasks using real-world data [36,37]. This work uses the same configuration as that presented by Clark et al. [36]. The network is trained either until a maximum of 50 epochs is reached or until validation loss does not decrease for 4 epochs.

## 3. Experimental Setup

Data are collected using a testbed setup consisting of a transmit-side system and a group of receive-side systems connected through a control and coordination back-plane. The testbed setup is reconfigurable for different collection scenarios and is the subject of a previous work, where its design, construction, and operation are presented in detail [38].

### 3.1. Transmit Hardware

The transmit-side hardware consists of a transmitter host computer and an interchangeable complement of 60 transmitters; this number was chosen to evenly distribute transmitters across each of the 12 USB hubs as described in [38] without causing undue strain on the USB ports of the transmitter host. The chosen transmitters are Great Scott Gadgets Yard Stick One (YS1) radios because of their USB connection, availability in large quantities, and similarity to many other commercial IoT transmitters. Officially a wireless test tool, these radios are capable of sub-1 GHz emissions with several modulation schemes at power levels up to 10 dBm. Each YS1 is connected to a 900 MHz 3 dBi omni-directional antenna. The transmitter host has a custom software and hardware arrangement to maximize the number of transmitters it can handle at one time. The core part of the machine is an Intel i7-12700 (8p+4e cores) processor with 64 GB RAM, 2 TB M.2 SSD, 16 TB SATA HDD, and a ST1000SPEX2 8P8C networking card. The front panel of the transmitter host has an array of 12 10-port USB 2.0 hubs, which are connected in groups of 4 to 3 PEXUSB3S44V USB 3.0 PCIe cards with independent host controllers. Each USB hub receives power from an auxiliary 300 W power supply through individual channels of a Numato Lab 16-channel USB relay. The chosen transmitters are prone to occasional failures that can only be recovered through a full power cycle and are automatically detected and resolved by toggling the power to the hub that hosts the failed device [38].

### 3.2. Receive Hardware

The receive-side hardware consists of 3 collection nodes (CNs), which are custom-built computers used for control, networking, and local data storage, and their associated software-defined radio (SDR). This number of CNs was chosen because this is the minimum number of collection nodes required to evaluate transfer learning performance between similar radios and different radios while collecting data simultaneously. To eliminate differences in multipath propagation between the radios, each shares a common 1.2 GHz 8 dBi flat patch antenna connected through a 3-way RF splitter network. Radios A and B were chosen to be USRP X310s with an SBX-120 (400–4400 MHz) daughterboard, and radio C was chosen to be a USRP B210 with its own integrated RF section based on the AD9361 transceiver. Each collection node has an Intel i5-11600K (6 cores) processor with 64 GB RAM, a 2 TB M.2 SSD for general use, a 14 TB SATA HDD for RF capture storage, and an Intel X520-DA2 SFP+ networking card.

### 3.3. Data Collection

Data collections are divided into individual runs of many transmissions conducted in series. Each transmission from a random YS1 of the available 60 is a randomized string of 1024 bytes at 31,250 baudwith a random modulation scheme at a center frequency of 915.25 MHz. The modulation scheme is any of the following: OOK, MSK, 4-FSK, 2-FSK, or GFSK. A log of each transmission payload, timestamp, the emitting device ID, and other metadata is stored in a ground-truth file for dataset labeling. When a transmission occurs, each receiver SDR captures the RF data simultaneously in the SigMF file format and includes the ground-truth information in the metadata. After each run, the positions of all radios in the USB hub array are scrambled to prevent any association between patterns of multipath propagation and any specific emitters. Runs are deliberately kept short to allow this scrambling to take place frequently.

Runs occur at a distance of 10 m with a direct line-of-sight path in an indoor environment with power levels of 10, 9, 8, 5, 0, and −10 dBm. Additionally, runs are conducted with a direct line-of-sight path outdoors at distances of 10 and 30 m.

## 4. Results and Analysis

**Figure 4:**
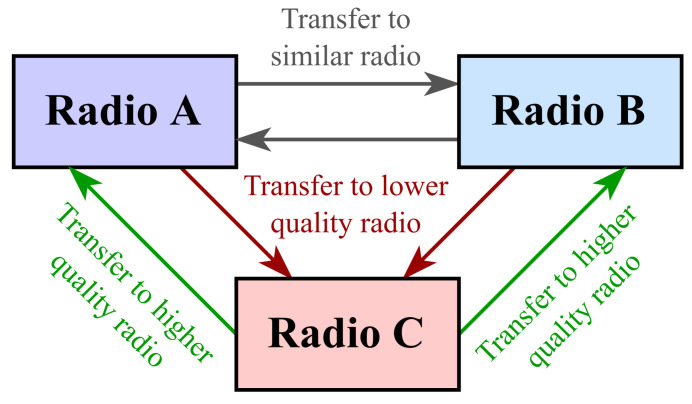
The six directions of transfer learning between similar and different radios grouped by the type of source and destination radios.

Results are split into four sections, namely baseline performance, transfer between similar radios (A → B and B → A), transfer to a lower-quality radio (A → C and B → C), and transfer to a higher-quality radio (C → A and C → B), as shown in Figure 4.

### 4.1. Expectations and Intuition

The focus of this work is comparing the performance disparity when transferring a model between data collected on similar radios and between data collected on dissimilar radios for two distinct spectrum-sensing applications. It has been observed in previous work that transferring AMC algorithms results in up to a 7% decrease in performance, even when using similar hardware [39]. It is anticipated that transferring from a relatively high-quality radio to a relatively low-quality radio will further decrease model performance. The same operation in the reverse direction, i.e., transferring a model trained on a relatively lower-quality radio to a relatively higher-quality radio, however, is expected to yield increased performance. This expectation is based on previous observations that training a model on high-SNR data and evaluating on low-SNR data led to lower performance, while the inverse case of training a model on low-SNR data and evaluating on high-SNR data actually led to increased accuracy [40]. Without further evidence in either direction, it is anticipated that this phenomenon generalizes to the overall reliability of the data, where low-SNR data and those collected on a low-quality receiver are considered less reliable, while high-SNR data and those collected on a high-quality receiver are considered more reliable.

The reasoning behind this expectation is based on the observed behavior of neural networks (NNs). An NN given high-quality data that are close to reality will be better able to observe and learn very subtle features associated with particular classes [41,42]. For the purposes of this discussion, the difference between high- and low-quality data is the relative frequency of receiver-based measurement errors from filtering, phase noise, thermal noise, I/Q imbalance, frequency offset, and timing drift [1,43]. This may lead to exceptionally high performance on data similar in quality and hardware distortions to the training data but will incur a penalty when asked to perform classification on comparatively lower-quality data, where it is harder to discern the subtle features that would otherwise be present in higher-quality data. An NN trained on lower-quality data will not have as much opportunity to learn such subtle features and, perhaps, will achieve slightly lower performance in its native environment but will incur no such performance penalty when operating on higher-quality data. In other words, it is expected that an NN trained on comparatively less precise RF data will be more general.

### 4.2. Baseline Performance

**Figure 5:**
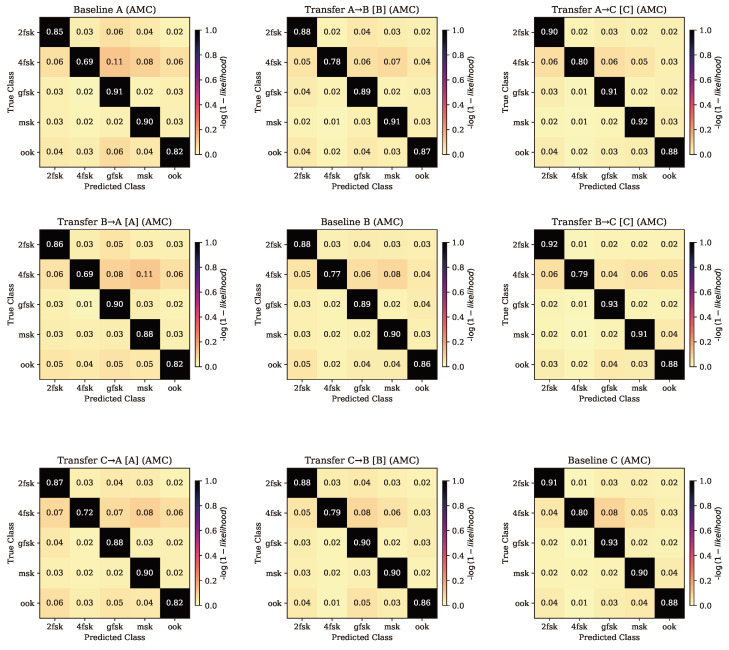
AMC confusion matrices for the baseline and transferred cases. Rows are organized by original radio. Columns are organized by evaluation radio (and transferred radio, if applicable). Baseline cases are on the diagonal, and TL cases are the other entries.

**Figure 6:**
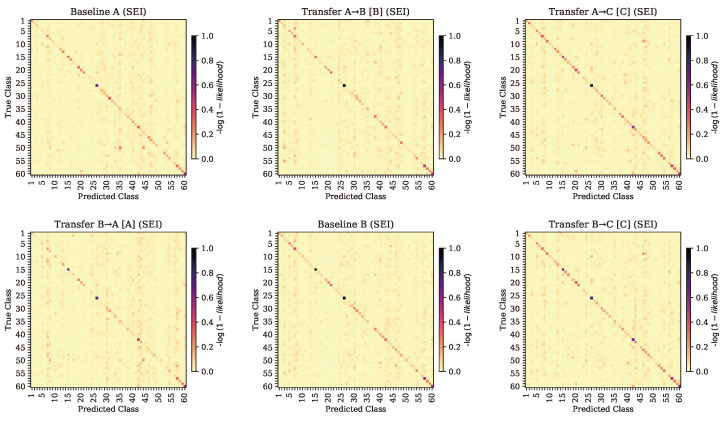

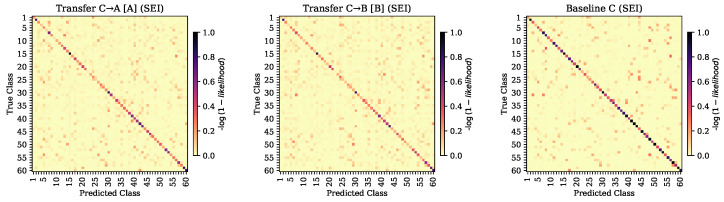
SEI confusion matrices for the baseline and transferred cases. Rows are organized by original radio. Columns are organized by evaluation radio (and transferred radio, if applicable). Baseline cases are on the diagonal, and TL cases are the other entries.

Once the baseline SEI and AMC models were trained on their respective training datasets, they were evaluated on a testing partition of the dataset, which they had not seen during the training process. In regards to SEI, the models followed the anticipated trends. The model trained on data from radio A performed best on data from radio A, with a slight performance drop for data from radio B (a radio of similar construction) and performed worst on the data from radio C (a radio of relatively lower quality). The SEI model trained on data from radio B showed a similar pattern, with the highest performance on its own dataset, slightly lower performance on data from the similar radio (radio A), and the lowest performance on data from radio C. The baseline SEI model for data from radio C performed best on dataset C and performed similarly poor on datasets A and B. The confusion matrices for the baseline models, with performance evaluated on data from their respective radios, is shown on the diagonals of Figure 5 and Figure 6.

**Figure 7:**
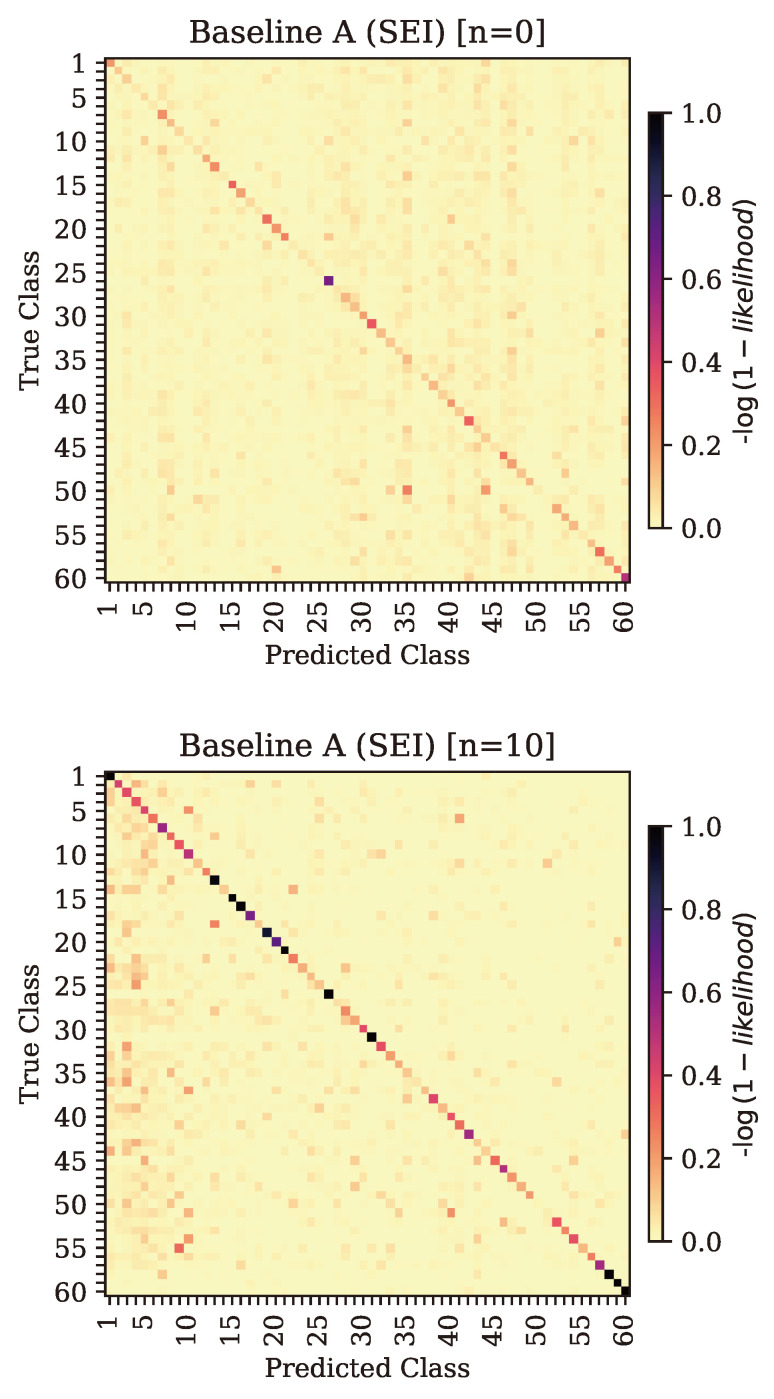

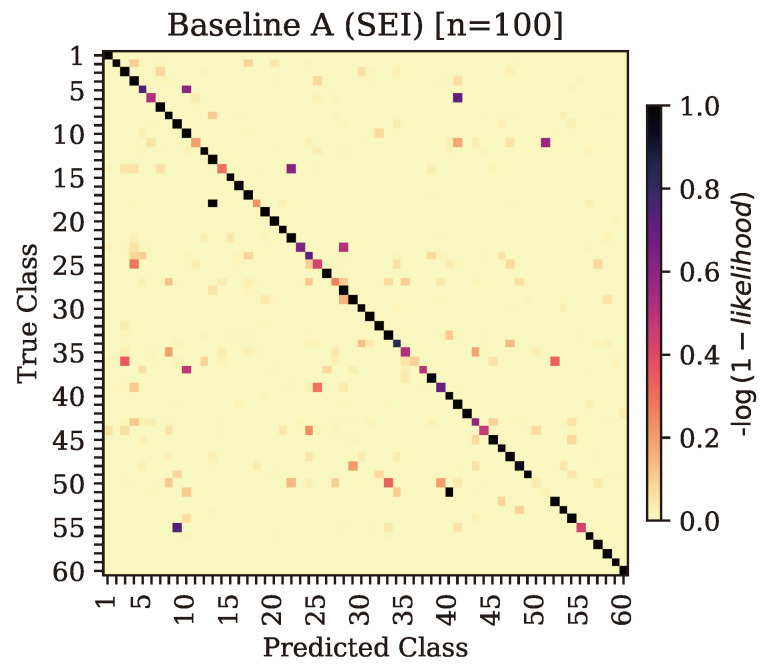
Confusion matrices of the baseline model for radio A with successive iterations of multinomial decision aggregation.

**Figure 8:**
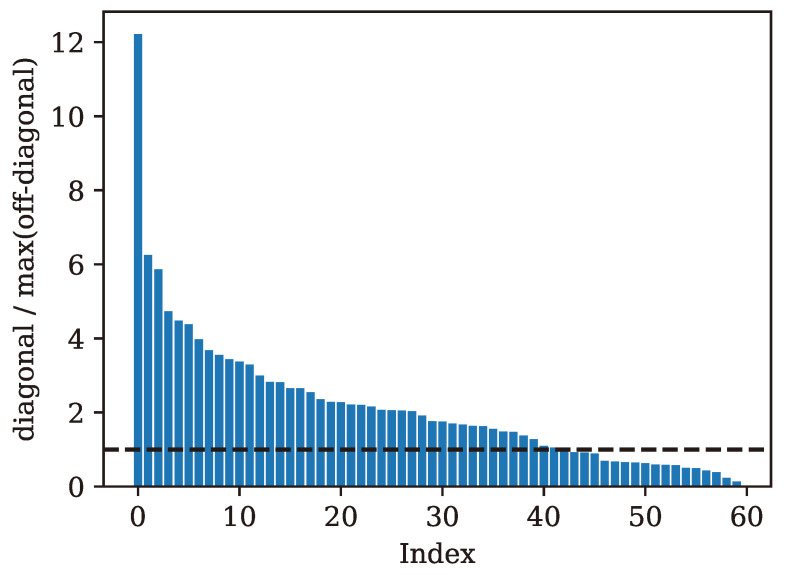
Ratios of diagonal entries (likelihood of correct classification) to maximum off-diagonal entries (highest likelihood of incorrect classification) of the confusion matrix of the baseline model for radio A. A dashed line separates ratios above 1, where classification performance tends to increase through successive aggregation iterations.

Table 2 shows the baseline classification accuracy of each model evaluated on each dataset, without any tuning. Of the three baseline models that were trained for SEI, the one with the best overall performance was the baseline model for the radio of lowest quality (radio C), which had a classification accuracy of around 46%. The baseline SEI classification accuracy for models trained on data from radios A and B was around 15%. It is important to note that, while these baseline accuracy numbers for SEI appear low, they represent performance on a 60-class classification problem. In recognizing that we can observe multiple successive inputs from the same source, the use of decision aggregation techniques such as multinomial-based aggregation [44] can offer significant improvements in overall performance, particularly when the performance is substantially higher than random guessing. To demonstrate the concept, the resulting confusion matrices of successive iterations of decision aggregation for baseline model A, the lowest-performing baseline SEI model, are shown in Figure 7. Through aggregation, the baseline classification SEI accuracy can be elevated to 33% after 10 iterations and 74% after 100 iterations. A histogram of ratios of the diagonal entries to the maximum off-diagonal entry for each true class is shown in Figure 8. Correct predictions tend to become more accurate over successive iterations of aggregation for classes where this ratio is above 1.

AMC classification accuracy was fairly consistent between models, with every model achieving the highest performance on dataset C and the lowest performance on dataset A, regardless of which dataset the model was original trained on.

### 4.3. Transfer between Similar Radios

When learned features from SEI model A were transferred and evaluated on dataset B and vice versa, it was expected that performance gains would be minimal, if present at all. As each of these datasets was created using a radio of the same quality, features learned by each model were expected to be consistent. Once TL was applied, it could be seen that these predictions held true, as can be seen in Table 3. Transferring model A’s features to a model that was further trained on dataset B produced modest SEI performance gains on dataset B, with an accuracy of 13%—up from the baseline performance of 9% on that dataset. However, this model proved to no longer perform as well on its original dataset (A), with accuracy dropping to 9% from the original 14%. This trend was mirrored when transferring from B to A, where performance on dataset A increased slightly to 11% from 10% but again dropped on its original dataset from 16% to 11%. The primary confusion matrices for these two cases are shown in entries (1,2) and (2,1) of Figure 6.

Regardless of the training and tuning process, classification accuracy for AMC remained consistent with the patterns observed for the baseline case; both models achieved consistently higher performance when evaluated on dataset B. The primary confusion matrices for these two cases are shown in entries (1,2) and (2,1) of Figure 5.

It should be noted that the TL models trained on a particular dataset consistently achieved the highest accuracy on test data taken from the most recently seen dataset, regardless of the baseline model used to transfer features, meaning model A transferred to dataset B performed best on test dataset B, when prior to TL, model A performed best on dataset A.

### 4.4. Transfer to Lower-Quality Radio

The performance of models trained on one type of radio, then transferred to a lower-quality radio, is shown in Table 4.

For SEI, transferring learned features from models created using data collected on higher-quality radios to a dataset captured on a lower-quality radio was predicted to produce a marginal decrease in performance compared to the baseline model but not a large enough decrease to render the model useless. It was instead found that when models A and B were transferred to a new model trained on dataset C, the models were able to outperform their baseline counterparts on dataset C, showing classification accuracies of 18% and 17%, respectively. This was an increase over their prior performance on dataset C in the baseline case and an increase over the performance on their original datasets. The SEI models’ abilities to identify radios in datasets A and B were both diminished, from a baseline 14% to 5% on dataset A and from a baseline performance of 16% to 5% for dataset B. The performance gains did not surpass the baseline performance of a model trained on dataset C; however, the transferred models’ abilities to classify examples from dataset C were significantly higher than those of the baseline models for datasets A and B, supporting the benefit of transfer learning in this case. The primary confusion matrices for these two cases are shown in entries (1,3) and (2,3) of Figure 6.

For AMC, performance remained consistently high on dataset C for both models. Performance remained slightly lower when evaluated on the other two datasets, regardless of the model. The primary confusion matrices for these two cases are shown in entries (1,3) and (2,3) of Figure 5.

### 4.5. Transfer to Higher Quality Radio

When transferring learned features from models generated using data collected on lower-quality radios to models trained on data collected by higher-quality radios, an increase in performance was expected, as the features learned by the lower-quality models were anticipated to be applicable to higher-quality data but not the reverse. It was found that when transferring the features, the models’ abilities to perform SEI on the dataset used for training were notably higher than those of the baseline model for those same datasets. AMC performance remained consistent, without seeing any significant increase or decrease in performance. The primary confusion matrices for these two cases are shown in entries (3,1) and (3,2) of Figure 5. The performance of models transferred from a lower- to a higher-quality radio is shown in Table 5.

The SEI model trained on dataset C, when further trained on examples from dataset A, achieved an accuracy of 30% on dataset A, while the baseline SEI model for dataset A was only able to achieve a classification accuracy of 14%. When transferred to dataset B, the model showed an SEI accuracy of 26% on dataset B, while the baseline model for dataset B only achieved an SEI accuracy of 16%. Each of the models, once transferred, showed a decrease in SEI accuracy when evaluated again on their origin datasets. The primary confusion matrices for these two cases are shown in entries (3,1) and (3,2) of Figure 6.

These results suggest that SEI models generated using data captured by lower-quality radios can be applied to small amounts of data captured from higher-quality radios to produce accuracy results superior to what could have been attained using only the data captured from the higher-quality radio.

### 4.6. Summary

For SEI, the baseline models, which did not utilize transfer learning, showed the strongest performance when tested on their respective test datasets. This result is to be expected, since the training and evaluation environments are the most similar. When transferred to a similar radio, performance improved for the dataset they were transferred to but decreased on the initial dataset. Even when performance decreased on the initial dataset, it remained greater than or equal to the performance on the other dataset seen before transfer. This implies that the model developed a level of generalization between radios that it was otherwise not capable of.

The high-to-low TL SEI models, which were initially trained on the higher-quality radios and fine-tuned on lower-quality radios, showed a similar pattern of increased performance on the lower-quality radios but decreased performance on their initial training sets. Notably, classification accuracy after transfer learning exceeded that achieved in the baseline case. Using only a small amount of tuning data from dataset C, the TL process increased classification accuracy from 6% to 18% for model A and from 5% to 17% for model B. The most intriguing result was from the trial involving the transfer of a model trained on a lower-quality radio to a higher-quality radio. The SEI models transferred in this manner were able to achieve significantly higher performance than the baseline model for the higher-quality radios.

The trends observed in the SEI data suggest that models for SEI perform well when they are forced to adapt and generalize across scenarios where data can be less reliable. When high-precision data are unavailable, the model is forced to search for a simpler fit to noisier data, reducing overfitting and eventually resulting in higher evaluation performance. These results suggest that the models trained on the lower-quality radios were underfitting but were still able to find larger correlations in the data. When eventually allowed to tune on higher-fidelity data, these models were able to achieve a much better fit. The two-step training process forced the model to focus on large trends first, then later hone in on important small details of the unique RF fingerprints. This is further supported by the high-to-low transfer not seeing the same level of performance gains, suggesting that the patterns learned on higher-fidelity data are not as applicable to classification on lower-fidelity data as the reverse case.

For AMC, performance remained consistent across the board, regardless of training or transferring process. This suggests that models are able to learn necessary features for AMC performance regardless of the source dataset, but in deployment, the source of these data still contributes to final performance.

## 5. Conclusions and Future Work

This work analyzed the performance implications of applying transfer learning for the purposes of heterogeneous platform adaptation to the RFML tasks of SEI and AMC. Radios of higher quality (USRP X310) and lower quality (USRP B210) were utilized to simultaneously collect data as they were transmitted by 60 identical radios hosted in a coordinated testing setup. The collected datasets of the same transmissions from both the higher- and lower-quality radios were used to train separate SEI and AMC models; then, transfer learning was utilized to apply learned features from each model to fine-tune new models intended for the counterpart dataset. This transfer learning method was able to produce models with significantly higher SEI classification performance on higher-quality radios than what was previously achievable using only data from that radio.

This work shows that TL techniques can be applied to RFML classification tasks with heterogeneous receivers to increase performance beyond what is typically achievable with a dataset from only one receiver. When TL is used to transfer features learned from a dataset from a lower-quality radio to a model then trained on a dataset from a higher-quality radio, a significant increase in performance can be gained. This is in contrast to the performance achievable with only a single training step on just the dataset from the higher-quality radio. The reverse, i.e., transferring features from a dataset from a higher-quality radio to a dataset from a lower-quality radio, produces a model with lower classification accuracy than simply collecting data and training on the lower-quality radio. Finally, transferring features learned by SEI models to new models using data collected from a similar radio does not seem to cause the model to undergo significant changes and results in only marginal performance increases. These findings prove that TL can be used to improve the performance of RFML classification models with access to varying-quality measurements of the same data. This method also allows for the re-use of weights of an existing model with a different radio without the need to collect enough data to fully train a new model.

When considering TL, an important point contrasting the tasks of AMC and SEI is the quantity of data necessary to train models capable of high performance. The 5-class AMC problem considered in this work proved relatively easy compared to the 60-class SEI problem; even with 12 times the data, the single-example classification performance for AMC was much higher than that for SEI. Given the much greater difficulty of SEI over AMC, a much larger quantity of examples per class may be required for acceptable large-class SEI performance, increasing the upfront cost of creating new SEI models from scratch and further making TL an attractive option when data on the desired platform are sparse.

To expand upon the completed experiment, one can look to determine the minimum quality of radio that can be used to collect transmissions that is still capable of training a model with features that are applicable to data collected on higher-quality radios. Future work could also include analysis of the features that are transferred to subsequent models and analyze the effects of training SEI and AMC models with weights that emphasize these features. This effort could result in a cheap and effective way to produce RFML models that are easily transferable to a wide variety of datasets, allowing for SEI and AMC models to be easily created and applied to large varieties of data.

## Figures and Tables

**Table 1:** Comparison of some existing RFML works utilizing transfer learning for classification.

Reference	Source	Task	Summary	Year
[17]	Circuits, Systems, and Signal Processing	SEI	Presents a method for radar signal identification by using transfer learning on a neural network architecture with an LSTM component. Transfer learning is used to reduce LSTM training requirements for classification of a set of devices not included in the original training set.	2020
[18]	MDPI Sensors	SEI	Proposes a method of embedding radar signals into a three-dimensional time–frequency–energy distribution for emitter identification. Uses transfer learning to increase robustness in the face of SNR and channel variation.	2016
[19]	Journal of Communications	SEI	Proposes a clustering and resampling method of improving similarity between training and deployment datasets. Transfers learned information about the structure of deployment data to optimize the performance gain from training.	2016
[20]	MDPI Information	SEI	Proposes a method of radar emitter identification with limited labeled offline training data. Uses a combination of transfer learning and online learning to improve model performance over time from a baseline support vector machine trained on offline data.	2019
[22]	IEEE Access	SEI	Proposes a method for classification of ADS-B and ACARS transmissions with a convolutional neural network. Uses transfer learning to extend the model’s capabilities to identify signals outside of the initial dataset.	2019
[23]	Security and Safety	SEI	Presents a specification of fine-tuning and distance metric protocols for transferable RF fingerprints for the purpose of IoT device authentication.	2024
[24]	IEEE Journal of Selected Topics in Signal Processing	AMC	Performs an in-depth study of various signal classification techniques. Includes analysis of transfer learning performance between synthetic and over-the-air domains.	2018
[25]	IEEE Transactions on Cognitive Communications and Networking	AMC	Proposes a technique for deep feature extraction of classifiers for known and unknown domains of signals. Uses transfer learning to adapt the algorithm to dynamic channel environments.	2024
[26]	IEEE Transactions on Vehicular Technology	AMC	Proposes a federated transfer learning framework between receivers attached to UAVs for distributed modulation classification in varying channel environments.	2024
[27]	IEEE Access	AMC	Presents a technique combining a graph convolutional network with long short-term memory cells in a single architecture for modulation recognition. Uses transfer learning to create a pre-trained model from an existing dataset that is later fine-tuned for higher performance on the desired dataset.	2024

**Table 2:** Baseline performance of models on each dataset.

Task	Model	Dataset A	Dataset B	Dataset C
	A	85%	87%	**90%**
AMC	B	85%	87%	**89%**
	C	84%	86%	**89%**
	A	**14%**	9%	6%
SEI	B	10%	**16%**	5%
	C	7%	3%	**46%**

**Table 3:** Transfer performance between similar radios.

Task	Model	Dataset A	Dataset B
AMC	A→B	84%	**87%**
	B→A	84%	**87%**
SEI	A→B	9%	**13%**
	B→A	**11%**	11%

**Table 4:** Performance when transferred to lower-quality radio.

Task	Model	Dataset A	Dataset B	Dataset C
AMC	A→C	84%	-	**89%**
	B→C	-	87%	**89%**
SEI	A→C	5%	-	**18%**
	B→C	-	5%	**17%**

**Table 5:** Performance when transferred to higher-quality radio.

Task	Model	Dataset A	Dataset B	Dataset C
AMC	C→A	**85%**	-	88%
	C→B	-	**87%**	89%
SEI	C→A	**30%**	-	14%
	C→B	-	**26%**	5%

## Data Availability

The data presented in this study are available on request from the corresponding author due to size constraints. The datasets presented in this article will be considered for separate publication after sponsor review. Requests to access the datasets should be directed to corresponding author.

## References

[1] Kulin M., Kazaz T., Moerman I., De Poorter E. (2018). End-to-End Learning From Spectrum Data: A Deep Learning Approach for Wireless Signal Identification in Spectrum Monitoring Applications. IEEE Access.

[2] Xu W., Trappe W., Zhang Y. Channel Surfing: Defending Wireless Sensor Networks from Interference. Proceedings of the 6th International Conference on Information Processing in Sensor Networks, Association for Computing Machinery.

[3] Dobre O. (2015). Signal Identification for Emerging Intelligent Radios: Classical Problems and New Challenges. IEEE Instrum. Meas. Mag..

[4] Xie S., Ye J. Overview of Automatic Modulation Recognition Methods. Proceedings of the 2023 International Conference on Distributed Computing and Electrical Circuits and Electronics (ICDCECE).

[5] Qu L.Z., Liu H., Huang K.J., Yang J.A. (2021). Specific Emitter Identification Based on Multi-Domain Feature Fusion and Integrated Learning. Symmetry.

[6] Tu Y., Lin Y., Hou C., Mao S. (2020). Complex-Valued Networks for Automatic Modulation Classification. IEEE Trans. Veh. Technol..

[7] Tsakmalis A., Chatzinotas S., Ottersten B. (2014). Automatic Modulation Classification for Adaptive Power Control in Cognitive Satellite Communications. Proceedings of the 2014 7th Advanced Satellite Multimedia Systems Conference and the 13th Signal Processing for Space Communications Workshop (ASMS/SPSC).

[8] Lin R., Ren W., Sun X., Yang Z., Fu K. (2020). A Hybrid Neural Network for Fast Automatic Modulation Classification. IEEE Access.

[9] Ramjee S., Yang D., Gamal A.E., Eldar Y.C. (2019). Fast Deep Learning for Automatic Modulation Classification. arXiv.

[10] Kulin M., Kazaz T., De Poorter E., Moerman I. (2021). A Survey on Machine Learning-Based Performance Improvement of Wireless Networks: PHY, MAC and Network Layer. Electronics.

[11] Sankhe K., Belgiovine M., Zhou F., Riyaz S., Ioannidis S., Chowdhury K. (2019). ORACLE: Optimized Radio clAssification through Convolutional neuraL nEtworks. Proceedings of the IEEE INFOCOM 2019-IEEE Conference on Computer Communications.

[12] Kuzdeba S., Robinson J., Carmack J. Transfer Learning with Radio Frequency Signals. Proceedings of the 2021 IEEE 18th Annual Consumer Communications & Networking Conference (CCNC).

[13] Blitzer J., McDonald R., Pereira F. Domain Adaptation with Structural Correspondence Learning. Proceedings of the 2006 Conference on Empirical Methods in Natural Language Processing-EMNLP ’06, Association for Computational Linguistics.

[14] Sharma M., Holmes M., Santamaria J., Irani A., Isbell C., Ram A. Transfer Learning in Real-Time Strategy Games Using Hybrid CBR/RL. Proceedings of the 20th International Joint Conferences on Artificial Intelligence (IJCAI).

[15] Wong L.J., Michaels A.J. (2022). Transfer Learning for Radio Frequency Machine Learning: A Taxonomy and Survey. Sensors.

[16] Pan S.J., Yang Q. (2010). A Survey on Transfer Learning. IEEE Trans. Knowl. Data Eng..

[17] Wang X., Zhang Y., Zhang H., Li Y., Wei X. (2020). Radio Frequency Signal Identification Using Transfer Learning Based on LSTM. Circuits Syst. Signal Process..

[18] Yang Z., Qiu W., Sun H., Nallanathan A. (2016). Robust Radar Emitter Recognition Based on the Three-Dimensional Distribution Feature and Transfer Learning. Sensors.

[19] Liu Z., Yang J.a., Liu H., Wang W. (2016). Transfer Learning by Sample Selection Bias Correction and Its Application in Communication Specific Emitter Identification. J. Commun..

[20] Feng Y., Cheng Y., Wang G., Xu X., Han H., Wu R. (2019). Radar Emitter Identification under Transfer Learning and Online Learning. Information.

[21] Wang Y., Gui G., Gacanin H., Ohtsuki T., Dobre O.A., Poor H.V. (2021). An Efficient Specific Emitter Identification Method Based on Complex-Valued Neural Networks and Network Compression. IEEE J. Sel. Areas Commun..

[22] Chen S., Zheng S., Yang L., Yang X. (2019). Deep Learning for Large-Scale Real-World ACARS and ADS-B Radio Signal Classification. IEEE Access.

[23] Shen G., Zhang J. (2024). Exploration of Transferable Deep Learning-Aided Radio Frequency Fingerprint Identification Systems. Secur. Saf..

[24] O’Shea T.J., Roy T., Clancy T.C. (2018). Over-the-Air Deep Learning Based Radio Signal Classification. IEEE J. Sel. Top. Signal Process..

[25] Zhang M., Tang P., Wei G., Ni X., Ding G., Wang H. (2024). Open Set Domain Adaptation for Automatic Modulation Classification in Dynamic Communication Environments. IEEE Trans. Cogn. Commun. Netw..

[26] Zhou Q., Wu S., Jiang C., Zhang R., Jing X. (2024). Over-the-Air Federated Transfer Learning Over UAV Swarm for Automatic Modulation Recognition in V2X Radio Monitoring. IEEE Trans. Veh. Technol..

[27] Suetrong N., Taparugssanagorn A., Promsuk N. (2024). Enhanced Modulation Recognition Through Deep Transfer Learning in Hybrid Graph Convolutional Networks. IEEE Access.

[28] Bu K., He Y., Jing X., Han J. (2020). Adversarial Transfer Learning for Deep Learning Based Automatic Modulation Classification. IEEE Signal Process. Lett..

[29] Xu Y., Li D., Wang Z., Guo Q., Xiang W. (2019). A Deep Learning Method Based on Convolutional Neural Network for Automatic Modulation Classification of Wireless Signals. Wirel. Netw..

[30] Wang Y., Gui G., Gacanin H., Ohtsuki T., Sari H., Adachi F. (2020). Transfer Learning for Semi-Supervised Automatic Modulation Classification in ZF-MIMO Systems. IEEE J. Emerg. Sel. Top. Circuits Syst..

[31] Meng F., Chen P., Wu L., Wang X. (2018). Automatic Modulation Classification: A Deep Learning Enabled Approach. IEEE Trans. Veh. Technol..

[32] Kumar Y., Sheoran M., Jajoo G., Yadav S.K. (2020). Automatic Modulation Classification Based on Constellation Density Using Deep Learning. IEEE Commun. Lett..

[33] Kacenjar S.T., Dant A., Neely R., Pham T., Solomon L., Rainey K. Performance assessment of a machine-learning-derived digital RF communication classifier. Proceedings of the Artificial Intelligence and Machine Learning for Multi-Domain Operations Applications II.

[34] Ujan S., Navidi N., Landry R. (2020). An Efficient Radio Frequency Interference (RFI) Recognition and Characterization Using End-to-End Transfer Learning. Appl. Sci..

[35] Kuzdeba S., Robinson J. Data-Centric Approaches to Radio Frequency Machine Learning. Proceedings of the MILCOM 2022–2022 IEEE Military Communications Conference (MILCOM).

[36] Clark W.H., Hauser S., Headley W.C., Michaels A.J. (2021). Training Data Augmentation for Deep Learning Radio Frequency Systems. J. Def. Model. Simul. Appl. Methodol. Technol..

[37] West N.E., O’Shea T. (2017). Deep Architectures for Modulation Recognition. Proceedings of the 2017 IEEE International Symposium on Dynamic Spectrum Access Networks (DySPAN).

[38] Muller B.P., Wong L.J., Clark W.H., Michaels A.J. (2023). A Real-World Dataset Generator for Specific Emitter Identification. IEEE Access.

[39] Sathyanarayanan V., Gerstoft P., Gamal A.E. (2023). RML22: Realistic Dataset Generation for Wireless Modulation Classification. IEEE Trans. Wirel. Commun..

[40] Al-Shawabka A., Restuccia F., D’Oro S., Jian T., Costa Rendon B., Soltani N., Dy J., Ioannidis S., Chowdhury K., Melodia T. (2020). Exposing the Fingerprint: Dissecting the Impact of the Wireless Channel on Radio Fingerprinting. Proceedings of the IEEE INFOCOM 2020-IEEE Conference on Computer Communications.

[41] Zhang J., Woods R., Sandell M., Valkama M., Marshall A., Cavallaro J. (2021). Radio Frequency Fingerprint Identification for Narrowband Systems, Modelling and Classification. IEEE Trans. Inf. Forensics Secur..

[42] Alhoraibi L., Alghazzawi D., Alhebshi R., Rabie O.B.J. (2023). Physical Layer Authentication in Wireless Networks-Based Machine Learning Approaches. Sensors.

[43] Goransson B., Grant S., Larsson E., Feng Z. (2008). Effect of Transmitter and Receiver Impairments on the Performance of MIMO in HSDPA. Proceedings of the 2008 IEEE 9th Workshop on Signal Processing Advances in Wireless Communications.

[44] Michaels A.J., Wong L.J., Torra V., Narukawa Y. Multinomial-Based Decision Synthesis of ML Classification Outputs. Proceedings of the Modeling Decisions for Artificial Intelligence.

